# Expression of Irisin/FNDC5 in Breast Cancer

**DOI:** 10.3390/ijms23073530

**Published:** 2022-03-24

**Authors:** Kamil Cebulski, Katarzyna Nowińska, Karolina Jablońska, Hanna Romanowicz, Beata Smolarz, Piotr Dzięgiel, Marzenna Podhorska-Okołów

**Affiliations:** 1 Division of Histology and Embryology, Department of Human Morphology and Embryology, Wroclaw Medical University, 50-368 Wroclaw, Poland; kamil.cebulski@umw.edu.pl (K.C.); karolina.jablonska@umw.edu.pl (K.J.); piotr.dziegiel@umw.edu.pl (P.D.); 2 Department of Pathology, Polish Mother Memorial Hospital-Research Institute, 93-338 Lodz, Poland; hanna-romanowicz@wp.pl (H.R.); smolbea@wp.pl (B.S.); 3 Division of Ultrastructural Research, Wroclaw Medical University, 50-368 Wroclaw, Poland; marzenna.podhorska-okolow@umw.edu.pl; 4 Department of Human Biology, Faculty of Physiotherapy, Wroclaw University of Health and Sport Sciences, 51-612 Wroclaw, Poland

**Keywords:** irisin, FNDC5, breast cancer, cancer, Ki-67, PGC-1α, metastases

## Abstract

Irisin is a myokine formed from fibronectin type III domain-containing protein 5 (FNDC5), which can be found in various cancer tissues. FNDC5 and irisin levels have been poorly studied in the tumor tissues of breast cancer (BC). The aim of this study was to determine the levels of irisin expression in BC tissues and compare them to clinicopathological factors and Ki-67 and PGC-1α expression levels. Tissue microarrays (TMAs) with 541 BC tissues and 61 samples of non-malignant breast disease (NMBD; control) were used to perform immunohistochemical reactions. *FNDC5* gene expression was measured in 40 BC tissue samples, 40 samples from the cancer margin, and 16 NMBD samples. RT-PCR was performed for the detection of *FNDC5* gene expression. Higher irisin expression was found in BC patients compared to normal breast tissue. FNDC5/irisin expression was higher in patients without lymph node metastases. Longer overall survival was observed in patients with higher irisin expression levels. FNDC5/irisin expression was increased in BC tissues and its high level was a good prognostic factor for survival in BC patients.

## 1. Introduction

Breast cancer (BC) is one of the most prevalent malignancies in women. Despite significant advances in the knowledge of carcinogenesis and the mechanisms of cancer progression, early detection and effective therapy remain major problems. BC is often detected in its advanced stages when the available treatment is not very effective [1]. There are many studies that have searched for new methods of BC treatment, e.g., by inhibiting kinases such as MARK4 [2,3]; however, there is still a need to search for new potential drugs and prognostic and predictive markers for BC. Recent studies on BC and other cancer types have indicated that irisin could be one such potential marker.

Fibronectin type III domain-containing protein 5 (FNDC5) is a transmembrane protein [4] and a prohormone from which a 112 aa peptide (irisin) is released. The molecular weight of FNDC5 ranges from 20–32 kDa. The deglycosylated form of irisin ranges from 12 to 15k Da. N-glycosylation has a crucial role in the biological activity of irisin [5]. Irisin was first detected in mouse skeletal muscle fibers in 2012 by Boström et al. [6]. The researchers found that the release of irisin from muscles occurred in response to physical activity. However, the expression of the *FNDC5* gene is controlled by a transcription factor known as peroxisome proliferator-activated receptor gamma coactivator 1 alpha (PGC-1α). However, to date, the mechanism by which irisin is released from the prohormone has not been explained [7,8,9]. Irisin has been shown to play a key role in the conversion of white adipose tissue to brown adipose tissue by increasing the expression of thermogenin (UCP1) in mitochondrial combs. The UCP1 protein is crucial in the adaptation of organisms to low temperatures. It causes the inhibition of adenosine triphosphate (ATP) production in the respiratory chain [8]. Previous studies have confirmed the presence of irisin in cardiomyocytes, adipose tissue, kidneys, blood vessel walls, and many other tissues [10]. There are also studies that have indicated the possibility of using irisin as a drug, e.g., in inhibiting the deterioration of cognitive functions in patients with Alzheimer’s disease [11]. Kim et al. [12] showed that αVβ5 integrin was a receptor for irisin in osteocytes, and that the protein could be involved in osteoclastogenesis and bone remodeling. It has been suggested that irisin binds to the receptor after transitioning to a dimeric form [12]. The association of irisin with bone remodeling may be related to metastases to the bone tissue.

Further studies have shown that increased irisin levels are found in malignant lesions, such as lung cancer, gastrointestinal cancer, ovarian cancer, clear cell renal cell carcinoma, laryngeal cancer, and osteosarcoma [13,14,15,16,17,18,19]. In our previous paper, we observed an association of FNDC5/irisin expression with the histological malignancy grade, tumor size, and lymph node metastases in non-small cell lung carcinoma (NSCLC) [20]. It is thought that increased irisin levels in malignant tumors may lead to local hyperthermia, resulting in protein denaturation and the inhibition of tumor growth by blocking ATP formation in the mitochondrial respiratory chain [21]. Other in vitro studies have indicated that increased irisin levels could lead to the inhibition of proliferation, migration, and the epithelial–mesenchymal transition (EMT) through the PI3K/AKT pathway in lung cancer cells [15]. In turn, the stimulation of cell proliferation and invasion by the same pathway was reported in a study on liver cancer [22]. In another study involving osteosarcoma, irisin inhibited proliferation, migration, and invasion by reversing the IL-6-induced EMT through the STAT3-Snail signaling pathway [23].

To date, there has been one study on the expression and significance of irisin in BC. Kuloglu et al. [16] performed immunohistochemical (IHC) studies on BC tissue and found that irisin levels were significantly higher in BC tissue compared to normal breast tissue. In turn, Gannon et al. [24] conducted a study using an in vitro model. They found that irisin increased the cytotoxic effect of doxorubicin in MCF-7 and MDA-MB-231 cell lines. They did not observe this result in the control MCF-10a cells. Other studies have assessed plasma irisin levels in BC patients. Pravatopoulou et al. [25] showed lower serum irisin levels in women with BC compared to healthy women. Panagiotou et al. [26] reported higher serum levels of irisin in patients with benign and malignant breast lesions compared to the serum levels in healthy women. They concluded that irisin was an independent predictor of metastasis. Another study reported decreased serum irisin levels in patients with bone metastases compared to those without metastases [27]. These different results may be due to the release of irisin into the blood by various tissues, not only the tissues affected by cancer. Therefore, the careful examination of irisin levels in BC seems to be of great importance.

Due to the different results obtained by the determination of serum irisin, the main aim of this study was to investigate its levels in the cancerous tissues of BC patients. On the basis of this, we determined the location and level of irisin expression in BC cells and non-malignant breast disease (NMBD; control). Moreover, the relationship between the irisin level and clinicopathological data was examined. Another aim of this study was to determine the correlation of the expression of the transcription factor PGC-1α and cell proliferation antigen Ki-67 with the level of irisin in BC. Irisin expression levels have never been assessed in such a large cohort of BC patients.

## 2. Results

### 2.1. Immunohistochemical (IHC) Detection of Irisin Expression in Tissue Microarrays (TMAs) with Different Breast Cancer Types

**Figure 1:**
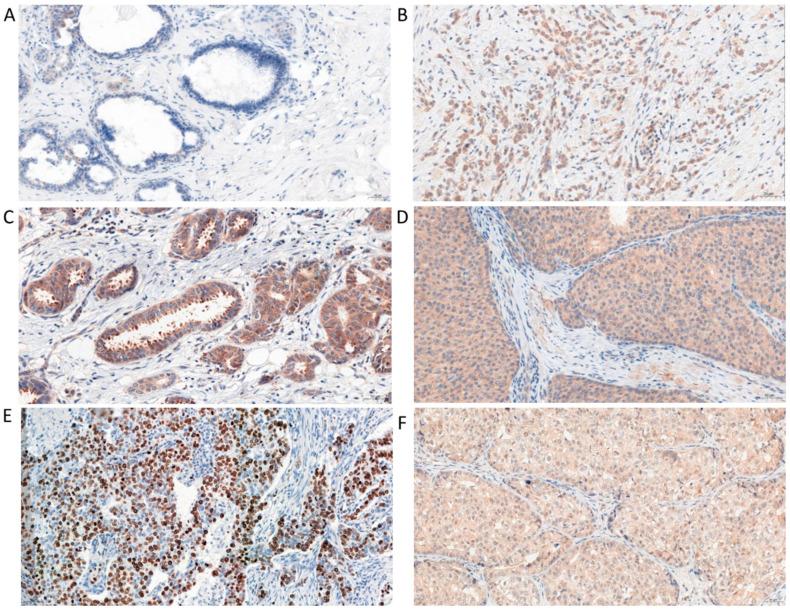
Comparison of irisin expression using immunohistochemistry (IHC; positive reactions—brown cell cytoplasm) in control tissue (**A**) and at different grades of malignancy (G) of breast cancer (BC; (**B**)—G1; (**C**)—G2, in apical parts of cells; (**D**)—G3) with Ki-67 (**E**) and PGC-1α (**F**); magnification x200.

**Figure 2:**
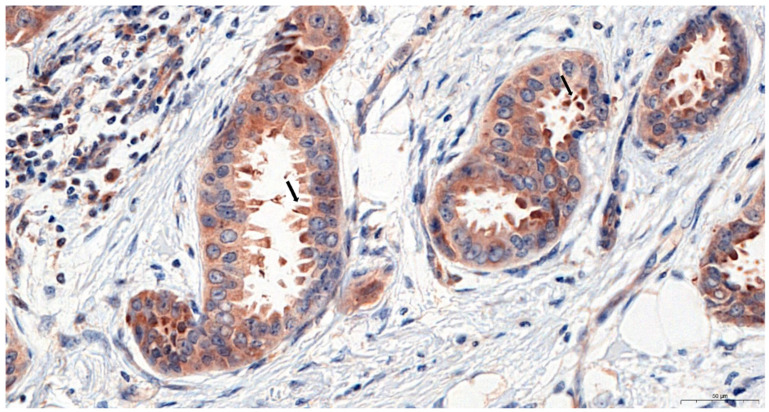
Positive immunohistochemical (IHC) reactions (brown color) indicating strong irisin expression in the apical parts of cancer cells and vesicles; magnification x400.

**Figure 3:**
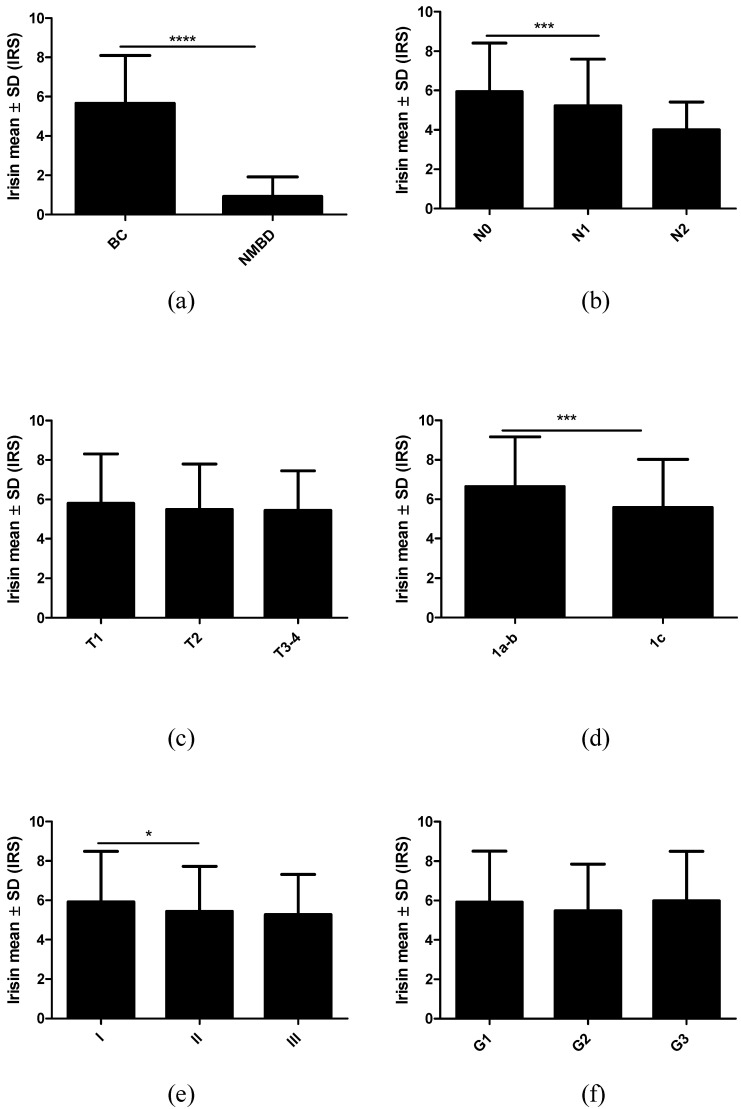
Comparison of irisin expression levels using immunohistochemistry (IHC) in breast cancer (BC) patients and control tissues (**a**) according to the lymph node status (**b**), tumor size (**c**,**d**), tumor stage (**e**), and malignancy grade (**f**). * p < 0.05, *** p < 0.001, **** p < 0.0001.

Irisin expression was found in the cytoplasm of BC cells, and a significantly lower level was reported in the NMBD samples. The expression level of this protein in BC (mean 5.7 ± 0.1 SE) was significantly higher than in NMBD samples (Mann–Whitney U test; p < 0.0001; mean 0.9 ± 0.3 SE); Figure 1A–D and Figure 3a). In addition, we observed increased irisin expression in the apical parts of tumor cells. We also reported the presence of irisin in vesicles secreted by tumor cells (Figure 2). However, we did not find irisin expression in the tumor stroma.

### 2.2. Associations between Irisin Expression in Cancer Cells and Clinicopathological Parameters

Table 1 shows the characteristics of BC patients with divided into high and low irisin expression levels according to the median. Table 2 shows the mean irisin expression levels in relation to several clinicopathological parameters. Figure 3 shows the results of the comparison of irisin expression levels in different groups of patients depending on clinicopathological features.

Irisin expression levels were lower in patients with lymph node metastases compared to patients without metastases (Mann–Whitney U test; p = 0.0002; Table 2, Figure 3b). Despite a decrease in irisin expression level with an increase in tumor size (T), this difference was not statistically significant (Figure 3c). However, we observed a statistically significant relationship between irisin expression in T1a-b compared to T1c (Mann–Whitney U test; p = 0.0002; Figure 3d). We also found higher irisin expression levels in tumors with a histological grade of G3 compared to those with a grade of G2 (Mann–Whitney U test; p = 0.0433; Figure 3f). Irisin levels decreased in more advanced stages of tumor progression. The difference between stage I and II was statistically significant (p = 0.0486; Figure 3e).

### 2.3. Associations between Irisin Expression and Overall Survival (OS)

**Figure 4:**
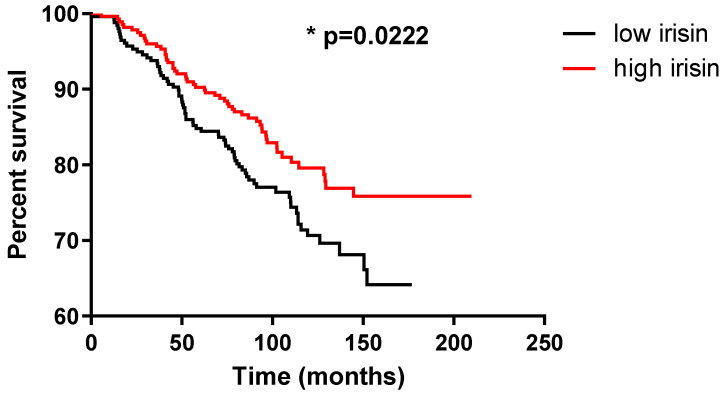
Kaplan–Meier survival curve presenting the prognostic impact of irisin expression levels using immunohistochemistry (IHC) on the overall survival (OS) of patients with breast cancer (BC). Patients were grouped according to the median value of irisin expression (5.3).

We found a relationship between irisin expression levels and overall survival (OS). Subjects with higher expression levels had a significantly longer survival time (Figure 4).

Overall survival time was also influenced by age, tumor size, lymph node metastases, histological grade, and stage. However, irisin expression level was not an independent prognostic factor (Table 3).

### 2.4. Comparison of the Expression Levels of Irisin with Ki-67 and PGC-1α

**Figure 5:**
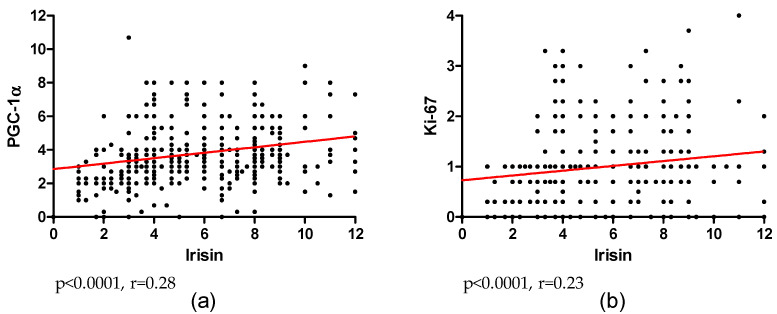
A moderate positive correlation of irisin expression level with the expression level of PGC-1α (**a**) and a weak correlation with Ki-67 antigen (**b**) in breast cancer (BC).

Due to the effect of the transcription factor PGC-1α on *FNDC5* gene expression, we analyzed its correlation with irisin levels in tumor cells. We found a moderate positive correlation (r = 0.28; p < 0.0001) between the expression levels of these two proteins in BC (Figure 5a). In addition, to investigate the association of irisin expression with increased BC cell proliferation, we evaluated its correlation with Ki-67 antigen expression. Both proteins were weakly positively correlated (r = 0.23; p < 0.0001; Figure 5b).

We did not find any statistically significant correlations between irisin expression levels and estrogen receptor (ER), progesterone receptor (PR), or HER2 status.

### 2.5. RT-PCR Detection of FNDC5 Gene Expression in BC and Control Tissue

**Figure 6:**
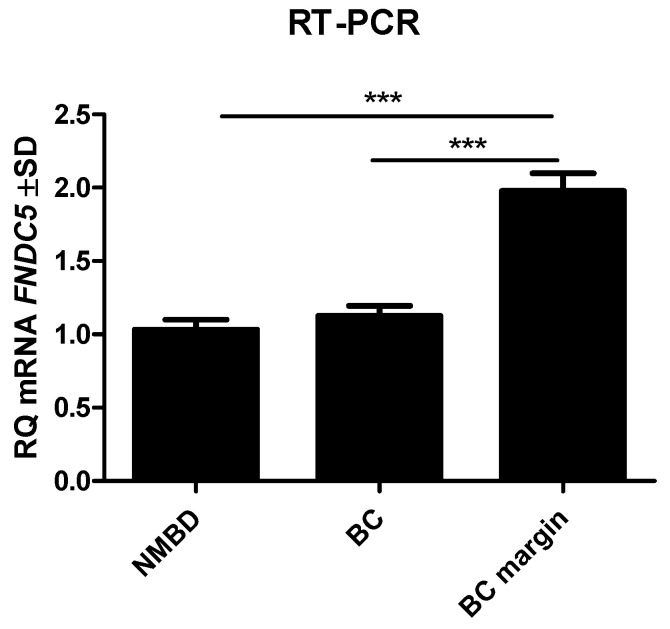
*FNDC5* gene expression in NMBD samples, breast cancer (BC) cells, and BC tumor margin. *** p < 0.001.

**Figure 7:**
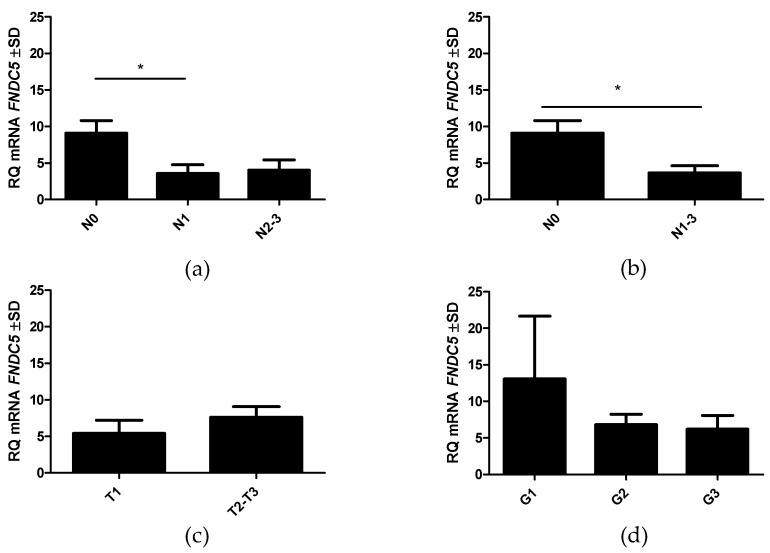
*FNDC5* gene expression in breast cancer (BC) cells according to the N status (**a**,**b**), tumor size (**c**), and malignancy grade (**d**). * p < 0.05.

The expression level of the *FNDC5* gene in BC was analyzed compared to the control samples (tissues from tumor margins and NMBD samples). The results indicated a significantly higher mRNA level in the tumor margin (mean 336.9 ± SD 70.12) compared to BC (mean 20.86 ± SD 22.71; Mann–Whitney U test p < 0.0001). No statistically significant difference was found in *FNDC5* mRNA expression in the NMBD samples compared to BC (mean 12.46 ± SD 7.1). However, *FNDC5* gene expression in the NMBD samples was significantly lower than that in the tumor margin tissue (Mann–Whitney U test p < 0.0001; Figure 6). As in the case of irisin levels assessed by IHC, we observed higher *FNDC5* gene expression in patients without lymph node metastases (mean 9.092 ± SD 1.7; p = 0.0395) compared to subjects with metastases (mean 3.679 ± SD 0.9; Figure 7a,b). We found no statistically significant difference in the *FNDC5* mRNA expression level with tumor size (T) or histological grade (G) (Figure 7c,d).

## 3. Discussion

Our study involved an analysis of irisin expression levels in BC in a large cohort of patients (*n* = 541). We demonstrated that FNDC5/irisin levels were higher in BC than in NMBD samples. In addition, we also found higher *FNDC5* mRNA levels in BC compared to NMBD samples.

The increased expression level of mRNA encoding FNDC5 in BC cells may be related to the metabolic changes occurring in the cancer cells. At the same time, the transcriptional coactivator PGC-1α, which stimulates the expression of irisin, has been implicated in many pathways that regulate metabolism and is related to mitochondrial biogenesis and UCP1, whose expression is stimulated by irisin and affects the uncoupling of the respiratory chain [6]. Thus, increased irisin levels may be a result of an increasing energy demand in cancer cells and a switch to glycolysis instead of obtaining ATP from the respiratory chain [18]. Irisin also increases glucose uptake by cells and enhances AMP-activated kinase (AMPK) phosphorylation [29]. Therefore, higher concentrations of irisin may allow cancer cells to obtain energy through glycolysis, which could confirm our findings related to the increased expression of irisin/FNDC5 in BC compared to the controls.

In some cases, we also found an increased concentration of irisin in the apical parts of cancer cells. Small vesicles showing the presence of irisin were often seen in the lumen of abnormal tubular structures in the tumor. This may be indicative of the apocrine secretion of irisin by some BC cells. Aydin et. al. [30] found irisin in breast milk, which indicates that it is also secreted into the ductal lumen under physiological conditions. Further studies are warranted to confirm the clinical significance of irisin secretion in BC patients.

The research conducted to date has mainly focused on evaluating plasma irisin levels in BC patients [25,27] and on in vitro studies [24]. The results have indicated lower serum irisin levels in BC patients than in control groups. Some researchers have suggested that an increase in the serum irisin level by one unit results in a 90% decrease in the risk of BC [18,25]. In our study, we obtained the opposite result. However, our study detected irisin in tissue samples and not in the serum of patients. This is probably the reason for the discrepancy between the results reported by Provatopoulou et al. [25] and our findings. Only Kuloglu et al. [16] focused on the immunohistochemical assessment of irisin in BC tissue samples. The authors found increased irisin levels in BC, ovarian, and cervical cells. Our results regarding irisin expression in BC are in line with the above studies. However, our study used TMAs, which indicated the expression level of irisin in the part of whole sections; this is a limitation of our study. However, studies comparing the results of IHC assessment on whole tissue material and TMAs indicate a very high accuracy of TMA in reflecting the whole tissue score [31,32]. In addition, compared to BC and NMBD samples, the higher *FNDC5* mRNA levels observed in normal tissue from the tumor margin are interesting. We supposed that the higher *FNDC5* mRNA level was associated with the interaction between healthy margin cells and cancer cells. In our previous study [33], we observed a higher expression of different markers in healthy tissues from the tumor margin compared to cancer cells. These changes were linked to epigenetic regulations. However, further studies are warranted to explain the cause of such a high level of *FNDC5* mRNA in the tissue from the surgical margin surrounding the tumor.

We also analyzed the association of irisin expression with clinicopathological factors. We demonstrated significantly lower levels of expression of *FNDC5* mRNA and FNDC5/irisin proteins in tumors of patients with lymph node metastases. A similar relationship was found for increasing tumor size (the difference was statistically significant only in the earliest stages, T1a–b vs. T1c) and for more advanced stages of the disease. Thus far, only Provatopoulou et al. [25] have shown a relationship between serum irisin levels and BC stage, tumor size, and lymph node metastases. However, they observed an inverse relationship compared to our findings. In their study, irisin levels increased with more advanced stages of the disease, a larger tumor size, and when lymph node metastases occurred. There are also noticeable discrepancies between our results and those of previous studies, which may be due to the fact that the mechanism of irisin secretion from FNDC5 into serum has not been understood yet. Moreover, serum irisin concentrations may be a result of irisin secretion by different tissues, such as adipose or muscle tissue. In our study, however, the level in actual BC tissue was analyzed.

We found that significantly lower irisin levels were associated with lymph node metastases (N1). In addition, lower *FNDC5* mRNA levels were observed in the group of patients with N1 cancer. This may suggest that high irisin levels prevent metastasis and cancer progression. Zhang et al. [27] analyzed BC patients without distant metastases and with spinal metastases. Their study indicated that serum irisin levels were higher in patients without metastases. They also noted that high serum irisin levels were associated with the absence of spinal metastasis [27]. Lymph node metastasis is a well-known negative prognostic factor [34]. However, there are currently no reports on the relationship between irisin levels in BC and lymph node and distant organ metastases. Research on other types of cancers has shown a similar relationship to that found in our study [18]. In our previous study [20], we observed a decrease in irisin levels in non-small cell lung carcinoma (NSCLC) cells from patients with lymph node metastases (N1) compared to those without metastases. We also reported a similar observation in laryngeal squamous cell carcinoma (LSCC) [19].

Cancer cells must undergo EMT for metastasis to occur. It has been suggested that higher irisin levels may inhibit EMT [15,18]. Our results showed higher irisin levels in the tumor tissue in the N0 group. This may indicate an inhibitory role of irisin in the EMT process of BC cells. Studies by Nowinska et al. [20], Pinkowska et al. [19], and Zhang et al. [27] support our hypothesis. Further studies are warranted to elucidate the mechanism of how irisin affects EMT inhibition and metastasis formation.

In our study, we also analyzed the survival of patients with low and high irisin levels in BC. We found that a lower irisin level was a negative prognostic factor. This finding indicates the beneficial effect of irisin in BC. In the study on NSCLC, we did not observe an association between the level of irisin expression in cancer cells and patient survival. We observed a longer overall survival time in patients with NSCLC only when irisin expression levels were lower in the tumor stroma [20]. However, in BC, FNDC5/irisin expression was not found in the tumor stroma when IHC was used. Nowinska et al. [20] suggested that high irisin expression in cancer-associated fibroblasts (CAFs) could be associated with its effect on the proliferation and EMT of tumor cells. In addition, irisin overexpression is suspected to have an inhibitory effect on cancer tumor growth through local hyperthermia and the dysregulation of ATP synthesis [16,18,21].

By analyzing the relationship between FNDC5/irisin expression and Ki-67, we showed that there was a weak positive correlation between them. The Ki-67 antigen is a well-known and widely used diagnostic marker of tumor cell proliferation [35]. The existence of a correlation between the proteins suggests that FNDC5 and irisin may affect BC cell proliferation to a limited extent. Panagiotou et al. [26] examined serum irisin levels in patients with benign and malignant breast cancer lesions and also observed a weak positive correlation with Ki-67 antigen expression. Pinkowska et al. [19] found a moderate positive correlation of irisin with Ki-67 expression in LSCC. Irisin in LSCC was also correlated with other proliferation markers, i.e., minichromosome maintenance protein 3 (MCM3) and metallothioneins I/II (MT-I/II) [19]. However, Wozniak et al. [36] reported a weak correlation between irisin and Ki-67, MCM3 protein, and urine diphosphate-galactose ceramide galactosyltransferase (UGT8) in colorectal cancer (CRC). In addition, some studies have indicated that in some cancers (e.g., hepatocellular carcinoma), irisin can enhance tumor cell proliferation by affecting the PI3K/AKT pathway [22]. Another study showed that high irisin expression levels could lead to the inhibition of proliferation, migration, and EMT [15]. In our study on NSCLC, we reported a dual effect of irisin on proliferation, depending on whether it was present in lung cancer cells or stromal cells. However, irisin expression in NSCLC cells correlated negatively with Ki-67 levels [20].

In this study, the results did not clearly indicate whether irisin has a beneficial effect on tumor cell proliferation. Irisin expression decreased with tumor size and disease progression, which is in contrast to a positive correlation of irisin expression with Ki-67. A partial explanation could be that irisin is responsible for the increase in UCP1 expression, which in turn leads to the inhibition of mitochondrial ATP synthesis and increases heat production [8,21]. Reduced cellular ATP levels are associated with AMPK activation and the inhibition of mTOR pathways. In turn, the AMPK-mTOR pathway is known to play a key role in regulating cancer cell proliferation [18,37]. This mechanism could explain why high levels of irisin expression in cancer cells are associated with the inhibition of tumor growth. It is possible that cells have increased irisin expression in the early stages of disease, which results in metabolic changes and affects mitochondrial biogenesis. In turn, in the later stages of the disease, irisin expression is suppressed due to its effects on UCP1 expression and the inhibition of ATP synthesis. A decrease in irisin expression with disease progression was also observed in our study. The differences between irisin expression levels and tumor size, especially tumors up to 1 cm (T1a–b) or > 1–2 cm in diameter (T1c), may be related to the progressive lack of oxygen and nutrients, and thus irisin production.

In conclusion, the assessment of irisin levels in BC may be useful to determine the stage of disease progression. We observed a decrease in irisin expression levels in BC. Moreover, high irisin levels were associated with longer survival. It seems important to verify whether the level of serum irisin reflects its level in tumor cells. Further studies are warranted to explain the mechanism of the effect of irisin on proliferation, migration, and EMT, and to test the possibility of using it as a target in potential therapy.

## 4. Materials and Methods

### 4.1. Patient Cohort

The archival samples included 541 paraffin blocks with sections of BC tissue that were obtained from the Polish Mother’s Memorial Hospital Research Institute between January 2004 and March 2012 in Lodz, Poland. The control samples included 61 NMBD samples obtained from the 4th Military Teaching Hospital in Wroclaw. A histological assessment was performed according to the World Health Organization criteria and the 8th TNM edition [28]. Table 1 shows the clinicopathological characteristics of the group of patients. Subjects who had undergone surgical treatment without radiotherapy or chemotherapy were enrolled in the study. In addition, frozen samples (−80 °C), including 40 BC and 40 control tissues obtained from tumor margins and 16 NMBD samples, were also used in the study. The patients whose material was used gave written informed consent. The study was approved by the Wroclaw Medical University Institutional Review Board and the Bioethics Committee (No. 726/2019 and KB-731/2019).

### 4.2. Tissue Microarrays (TMAs)

Twenty tissue microarrays (TMA) were prepared from 541 paraffin blocks with BC tissue sections. Two TMAs were also prepared from NMBD samples (control). For this purpose, the histological preparations were made from archival samples which were stained with hematoxylin and eosin. The slides were scanned using a Panoramic Midi II histological scanner (3DHISTECH Ltd., Budapest, Hungary). Cancer sites with a core size of 1.5 mm were selected by the Panoramic Viewer (3DHISTECH Ltd.) and were transferred to the tissue arrays using the TMA Grand Master (3DHISTECH Ltd.).

### 4.3. Immunohistochemical (IHC) Reactions 

IHC reactions were carried out on TMAs using primary antibodies to detect the expression of the proteins. Paraffin sections with a 4 μm thickness were prepared. After deparaffinization and hydration, thermal epitope demasking was performed using a Dako PT Link (Dako, Glostrup, Denmark) apparatus and low pH Target Retrieval Solution (Agilent Technologies, Santa Clara, CA, USA) for 30 min at 97 °C. To visualize the antigen, we used polyclonal rabbit anti-irisin (dilution 1:50; code no. NBP2-14024; Novus Biologicals, Littleton, CO, USA) with EnVision™ FLEX+ Mouse LINKER (Dako). Monoclonal mouse anti-Ki-67 antibodies (ready to use; clone MIB-1; code no. IS626; Dako) and polyclonal rabbit anti-PGC-1α antibodies (dilution 1:3200; code no. NBP1-04676, Novus Biologicals) were used to detect other markers. An automatic DAKO Autostainer Link 48 system (Dako) was used for the IHC reactions and an EnVision FLEX kit (Dako) was used to visualize the antigens. The slides were additionally stained with Mayer’s hematoxylin.

### 4.4. Assessment of IHC

The assessment of IHC reactions was performed by two independent investigators (PD and KC). The evaluation was performed using a BX41 light microscope (Olympus, Tokyo, Japan) coupled with the CellD software (Olympus). The cytoplasmic expression of irisin and PGC-1α was assessed using the semiquantitative method immunoreactive score (IRS) according to Remmele and Stegner [38]. The final result was the product of the scores obtained by the estimation of intensity (1—weak, 2—moderate, or 3—strong) and the percentage of cancer cells with a positive reaction (0—no expression, 1 point—> 1%–≤ 10%, 2 points—>10 –≤ 50%, 3 points—> 50%–≤ 80%, 4 points—> 80%). A five-point evaluation scale was used to assess the nuclear expression of Ki-67 (0—no expression, 1 point—> 1%–≤ 10%, 2 points—> 10%–≤ 25%, 3 points—> 25%–≤ 50%, 4 points—> 50%) [20].

### 4.5. Real-Time PCR (RT-PCR)

Material preserved in RNAlater (Thermo Fisher Scientific, Massachusetts, WT, USA) was used for RT-PCR. The material consisted of 40 BC sections, 40 control tissues taken from the tumor margin, and 16 NMBD samples. RNA isolation was performed using an RNeasy Mini Kit (Qiagen). To obtain cDNA, a High-Capacity cDNA Reverse Transcription Kit with RNase Inhibitor (Applied Biosystems, Waltham, MA, USA) was used. The expression level of *FNDC5* (*FNDC5*; TaqMan Gene Expression Assay, Applied Biosystems) was tested using a 7900HT Fast Real-Time PCR System (Applied Biosystems) and the relative quantification (RQ) method. The analysis of *FNDC5* gene expression was performed using the RQ Manager 1.2 software (Applied Biosystems). The results were standardized in relation to the expression of the reference gene β-actin (ACTB; TaqMan Gene Expression Assay, Applied Biosystems). The expression level of the *FNDC5* gene in BC cells was assessed in relation to its level in the normal tissue. The analysis was repeated three times.

### 4.6. Statistical Analysis

Kruskal–Wallis, Mann–Whitney U, and chi-square tests were used to assess the association of irisin expression level with clinicopathological factors. Patient OS was measured from the day of surgery to death or to the last follow-up. Kaplan–Meier analysis and the log-rank test were used to examine the association of patient survival with irisin expression levels. The Cox proportional hazard regression model was used to evaluate the association of OS with the clinicopathological evaluation of patients (hazard ratio and 95% confidence intervals). The statistical significance of the difference in *FNDC5* mRNA expression in BC and normal tissue was assessed using the Mann–Whitney U test. Spearman’s rank correlation was used to test the correlation between the expression levels of irisin, Ki-67 antigen, PGC-1α, ER, PR, and HER2. A *p* value ≤ 0.05 was considered statistically significant. Statistical analysis was performed using Prism 5.0 (GraphPad, La Jolla, CA, USA) and Statistica 13.1 (StatSoft, Cracow, Poland).

## Figures and Tables

**Table 1:** Characteristics of BC patients related to low and high expression of irisin (divided according to the median value of 5.6) compared with chi-square tests.

Clinicopathological Parameter	*n* 541 (%)	Irisin Expression in BC Cells		
		Low >0–<5.6	High ≥5.6–12	Chi^2^ Test *p* Value
**Age ≤ 60**	156 (28.8)	75 (48.1)	81 (51.9)	0.0707
**>60**	385 (71.2)	218 (56.6)	167 (43.4)	
**Histology type IDC**	521 (96.3)	281 (53.9)	240 (46.1)	0.7252
**ILC**	12 (2.2)	7 (58.3)	5 (41.7)	
**IPC**	1 (0.2)	1 (100.0)	0 (0.0)	
**MC**	1 (0.2)	0 (0.0)	1 (100.0)	
**MetC**	4 (0.7)	3 (75.0)	1 (0.0)	
**MucC**	2 (0.4)	1 (50.0)	1 (50.0)	
**Tumour size (T) T1**	344 (63.6)	183 (53.2)	161 (46.8)	**0.0414**
**[T1a-b**	[89 (16.5)	35 (39.3)	54 (60.7)	
**T1c]**	255 (47.1)]	148 (58.0)	107 (42.0)	
**T2**	179 (33.1)	99 (55.3)	80 (44.7)	
**T3-4**	18 (3.3)	11 (61.1)	7 (38.9)	
**Lymph nodes (N) N0**	340 (62.8)	169 (49.7)	171 (50.3)	**0.0258**
**N1**	193 (35.7)	119 (61.7)	74 (38.3)	
**N2**	8 (1.5)	5 (62.5)	3 (37.5)	
**Stage I**	237 (43.8)	123 (51.9)	114 (48.1)	0.5507
**II**	283 (52.3)	157 (55.5)	126 (44.5)	
**III**	21 (3.9)	13 (61.9)	8 (38.1)	
**Grade of malignancy (G)**				
**G1**	96 (17.7)	49 (51.0)	47 (49.0)	0.2929
**G2**	350 (64.7)	198 (56.6)	152 (43.4)	
**G3**	95 (17.6)	46 (48.3)	49 (51.7)	

Abbreviations: BC—breast cancer; IDC—invasive ductal carcinoma; ILC—invasive lobular carcinoma; IPC—invasive papillary carcinoma; MC—medullary carcinoma; MetC- metaplastic carcinoma; MucC—mucinous carcinoma.

**Table 2:** Association of irisin expression level with clinicopathological characteristics according to the World Health Organization criteria [28] in patients with BC; significance in bold.

BC	*p* Value (Mann–Whitney U Test)		Mean ± SD
**Lymph Nodes (N)**		**Lymph Nodes**	
**N0 vs. N1** **N0 vs. N2** **N1 vs. N2** **N0 vs. N1-2**	**0.0003** 0.2259 0.7913 **0.0002**	**N0** **N1** **N2**	5.9 ± 2.5 5.2 ± 2.4 4.9 ± 1.8
**Tumor** **Size (T)**		**Tumor size**	
**T1 vs. T2**	0.2864	**T1**	5.8 ± 2.5
**T1 vs. T3–4**	0.4505	**T2**	5.5 ± 2.3
**T2 vs. T3–4**	0.7400	**T3–4**	5.2 ± 2.0
**Tumor T1 size**		**Tumor T1 size**	
**T1a–b vs. T1c**	**0.0002**	**T1a–b** **T1c**	6.7 ± 2.5 5.5 ± 2.5
**Stage (S)**		**Stage**	
**I vs. II**	**0.0486**	**I**	5.9 ± 2.6
**I vs. III**	0.2121	**II**	5.5 ± 2.3
**II vs. III**	0.6178	**III**	5.2 ± 1.9
**Grade of malignancy (G)**		**Grade of malignancy**	
**G1 vs. G2**	0.0951	**G1**	6.0 ± 2.5
**G1 vs. G3**	0.8179	**G2**	5.5 ± 2.4
**G2 vs. G3**	**0.0433**	**G3**	6.0 ± 2.5

Abbreviations: vs.—versus.

**Table 3:** Univariate and multivariate Cox proportional hazards analyses in 541 patients with BC.

Clinicopathological Parameter	Univariate Analysis HR (95% CI) p	Multivariate Analysis HR (95% CI) p
**Age**	**3.11 (2.16** **–4.47)**	**2.45 (1.70** **–3.52)**
**≤60 vs. >60**	**<0.0001**	**<0.0001**
**pT**	**10.86 (3.65** **–32.35)**	1.67 (0.61–4.47)
**T1–T2 vs. T3–T4**	**<0.0001**	0.3098
**pN**	**3.02 (2.12** **–4.28)**	**2.10 (1.45** **–3.03)**
**N0 vs. N+**	**<0.0001**	**<0.0001**
**Grade**	**2.08 (1.38** **–3.12)**	**2.31 (1.16** **–4.60)**
**G1 vs. G2–G3**	**0.0005**	**0.0156**
**Stage**	**22.44 (7.73** **–65.16)**	2.06 (0.84–5.05)
**I–II vs. III–IV**	**<0.0001**	0.1155
**Irisin**	**0.68 (0.48** **–0.95)**	0.88 (0.61–1.30)
**≤5.6 vs. >5.6**	**0.0300**	0.5613
**Ki-67**	1.05 (0.51–2.21)	
**<25 % vs.** **≥25 %**	0.8773	
**PGC1** **α**	0.83 (0.57–1.17)	
**≤3.7 vs. >3.7**	0.3026	
**HER2**	1.56 (0.97–2.48)	
**0 vs. 1–3**	0.0644	
**PR**	0.71 (0.51–1.00)	
**0 vs. 1–3**	0.0596	
**ER**	0.76 (0.53–1.07)	
**0 vs. 1–3**	0.1272	

Abbreviations: HR—hazard ratio; CI—confidence interval; BC—breast cancer. Significance in bold.

## Data Availability

The raw data and the analytic methods will be made available to other researchers for purposes of reproducing the results in their own laboratories upon reasonable request. To access protocols or datasets contact katarzyna.nowinska@umw.edu.pl.
